# Molecular Characterization of Hetero-Pathogenic and Diarrheagenic *Escherichia coli* Pathotypes in Diarrheic Children under Five Years and Exposure Environment in Ogun State, South-West Nigeria

**DOI:** 10.3390/pathogens12111358

**Published:** 2023-11-15

**Authors:** Tosin Segun Ogunbiyi, Olanrewaju Emmanuel Fayemi, Gabriel Bidemi Akanni, Christianah Idowu Ayolabi, Tine Hald

**Affiliations:** 1 Department of Biological Sciences, Mountain Top University, Ibafo, 110106 Ogun State, Nigeria; gbakanni@mtu.edu.ng (G.B.A.); cayolabi@unilag.edu.ng (C.I.A.); 2 Department of Microbiology, University of Lagos, Akoka, 101017 Lagos State, Nigeria; 3 National Food Institute, Technical University of Denmark, 2800 Kongens Lyngby, Denmark; tiha@food.dtu.dk

**Keywords:** diarrhea, diarrheagenic *E. coli*, children, risk factors, hetero-pathogenic

## Abstract

Background: Diarrheagenic *Escherichia coli* (DEC) is one of the most common etiological agents of moderate-to-severe diarrhea in Low- and Middle-Income Countries (LMICs). Therefore, determining the source(s) of DEC in index cases and exposure environment is important for developing a prevention strategy. The current study aims to investigate the prevalence of DEC among children under 5 years and their exposure environment in Ogun State, Nigeria. Methods: Samples from 228 diarrheic children and their exposure environment were collected and screened for *E. coli*. Bio-chemically compatible distinct colonies were molecularly characterized using a 7-virulence-gene multiplex PCR with virulence factors (VFs) indicative of four pathotypes of *E. coli*: enterotoxigenic (ETEC), verotoxigenic (VTEC), enteropathogenic (EPEC), and enteroinvasive (EIEC). Representative pathotypes were subjected to antimicrobial susceptibility and over-expressed efflux pump assays. Results: One or more VFs typical of specific pathotypes were detected in 25.9% (59/228) diarrhea cases consisting of ETEC (21.5%) and EPEC (0.4%), while hetero-pathogenic pathotypes were found in 4.0% of cases. Of the food sources, 27.9% (101/362) were positive for DEC, of which ETEC accounted for 21.0%, VTEC 1.9%, EPEC 0.6%, EIEC 0.6%, and hetero-pathogenic pathotypes were 3.9%. Furthermore, ETEC was the only pathotype detected in the wastewater (4/183). Interestingly, the consumption of street-vended foods was the most significant (*p* = 0.04) risk factor for DEC infection in the study area. A total of 73.3% of selected DEC pathotypes showed resistance to antimicrobials, while 27.5% demonstrated over-expression of efflux pump activity. Conclusion: The high prevalence of ETEC across all sources and the occurrence of hetero-pathogenic DEC in diarrheic children and food sources emphasizes the importance of establishing a better strategy for the control and prevention of diarrhea among children in low- and medium-income households.

## 1. Introduction

Diarrhea is one of the leading causes of death in children under 5 years, accounting for an annual 1.7 billion global cases with an estimated mortality of 1.3 million deaths, with approximately 25% of this observed mortality occurring in Low- and Middle-Income Countries (LMICs) [1]. The number of diarrhea-related infant mortality in Nigeria remains high despite concerted global efforts aimed at reducing its incidence [2]. In Nigeria, the prevalence of diarrhea was estimated at 18.8%, with an annual mortality of 150,000 deaths [3]. Common routes of exposure to diarrhea are usually through consumption of contaminated water, unhygienic practices in food preparation, lack of potable drinking water, and improper disposal of sewage [4,5]. Different infectious agents have been implicated in the etiology of diarrheal diseases [4].

Diarrheagenic *Escherichia coli* (DEC) is one of the most common etiological agents of moderate-to-severe diarrhea in LMICs [6]. Various DEC strains are responsible for 30% to 40% of diarrhea episodes in children in developing countries [7]. The DEC group has recently been classified into nine distinct pathotypes based on pathogenomics, phenotypic classification and essential virulence genes defining each subgroup, such as Shiga toxin-producing *E. coli* (STEC), enterohemorrhagic *E. coli* (EHEC), enteropathogenic *E. coli* (EPEC), enterotoxigenic *E. coli* (ETEC), enteroinvasive *E. coli* (EIEC), enteroaggregative *E. coli* (EAEC), diffusely adhering *E. coli* (DAEC), adherent-invasive *E. coli* (AIEC), and cell-detaching *E. coli* (CDEC) [8,9]. The EPEC, ETEC, and EAEC are the most common DEC pathotypes and are known to cause moderate-to-severe diarrhea or even death in children in Asia and Africa [10,11,12]. However, studies have reported DEC strains that harbor combinations of virulence factors that are characteristic of two or more DEC pathotypes. These are called hetero-pathogenic *E. coli* [13]. Previously reported combinations of virulence factors that could lead to more severe diseases are EAEC/STEC, EPEC/STEC, and EPEC/ETEC [14,15,16]. Acute watery and bloody diarrhea cases with more severe symptoms caused by hetero-pathogenic strains in children under 5 years have been reported in Asia [15]. The prevalence and distribution of DEC as a cause of diarrhea in children under 5 years have been shown to vary within regions and countries [17,18,19,20]. Cases of hetero-pathogenic DEC consisting of EPEC/ETEC have been reported in Africa, specifically in Kenya, Ethiopia, and The Gambia [6,10,21]. However, there is a paucity of information on the different DEC pathotypes, most importantly hetero-pathogenic groups in children under 5 years in Nigeria. This study investigated the molecular epidemiology of hetero-pathogenic and diarrheagenic *E. coli* pathotypes and associated risk factors that predispose children under 5 years with diarrhea to DEC infection in South-West Nigeria.

## 2. Materials and Methods

### 2.1. Inclusion and Exclusion Criteria for Study Participation

Children of both sexes under the age of 5 years presenting with diarrhea and without any prolonged illnesses were enrolled for this research. Children with diarrhea, prolonged illnesses (even when under five years old), and older than 5 years were excluded from this study.

### 2.2. Study Location/Population

This study was a cross-sectional hospital-based study conducted between July 2020 and February 2022 at the paediatric wards of six hospitals: Sacred Heart Hospital (7°10′00.2″ N 3°21′20.2″ E), Mowe Primary Healthcare Centre (PHC) (6°48′28.7″ N 3°26′07.9″ E), Ibafo Health Clinic (6°44′38.2″ N 3°25′16.7″ E), Ofada PHC (6°51′54.7″ N 3°25′39.4″ E), Mokoloki PHC (6°52′55.8″ N 3°22′04.8″ E), and Federal Medical Centre (7°08′44.7″ N 3°22′41.4″ E). These hospitals are known to receive high number of paediatric patients across the state. In addition, the demography for this research was noted for low-middle-income earners and high population density. Diarrheal outpatient children ≤ 5 years of age attending these hospitals and whose parents consented to participation in the research were enrolled.

### 2.3. Sample Collection

Diarrheal stool samples were collected with the help of trained nursing staff. Briefly, samples were collected by allowing the diarrheal child to pass stool on a nylon wrap placed over the rim of a plastic potty or in a disposable diaper. Then, a sterile plastic spoon was used to transfer the stool to a clean, sterile universal bottle prefilled with Cary Blair transport medium (CM0519B, Oxoid, Basingstoke, UK). Sample collection was carefully performed to avoid contamination with urine, soil, or water. In addition, samples from food sources (left-over foods, drinking water, fruits, and vegetables) were fed to the child, and wastewater samples from the households of the diarrheic child were also collected in a clean, leak-proof container. Fruits and vegetables that could not be obtained from the households were bought from markets in proximity to the index cases. All samples were labelled, packed, sealed, and sent to the laboratory within 24 h for analyses. Interviewer-administered questionnaires were used to obtain information about clinical manifestations and sociodemographic characteristics, including gender, age, occupation (of the mother), and sanitary wares of the diarrheic child from the parent(s). The data obtained were used to explore risk factors for DEC-associated diarrhea in children within the study population.

### 2.4. Bacteria Isolation

Standard culture methods were performed on all samples immediately after reception in the laboratory. The isolation of a single *E. coli* colony was performed by plating the samples onto sorbitol MacConkey agar (Oxoid, Basingstoke, UK) and incubating for 24–48 h at 37 ± 1 °C. Determination of *E. coli* isolates was carried out using the following biochemical tests: carbohydrates fermentation, gas production, hydrogen sulphide production, citrate utilization, urease production, indole, and motility. Isolates positive for lactose–glucose and gas production, non-hydrogen sulphide-producing, negative citrate and urease, positive indole, and motility were ascribed as *E. coli*. The *E. coli* strain ATCC25922 was used as a positive control. After identification, pure *E. coli* isolates were stored in brain heart infusion broth (CM0225, Oxoid, Hampshire, UK) with 20% glycerol and stored at −80 °C before the molecular analyses the next day.

### 2.5. DNA Extraction

The DNA of a single *E. coli* colony was extracted following a method previously described by Choo et al. [22] with slight modifications. Briefly, 1 mL of the pure *E. coli* isolate-BHI culture broth was aliquoted into 2 mL snap vials, centrifuged at 5000× *g* for 3 min, the supernatant discarded, and the cell pellet resuspended in 1 mL sterile distilled water, pulse-vortexed, centrifuged, and the supernatant discarded. The cell pellets were resuspended in 200 µL double-distilled water and heated at 100 °C for 15 min in a dry heat block (Techne, UK), after which the vials were immediately cooled on ice for 5 min. Then, they were centrifuged at 8000× *g* for 7 min, and 100 µL of the supernatant was used as template DNA for subsequent PCR.

### 2.6. Detection of Virulence Factors for DEC

Multiplex PCR for *E. coli* virulence genes using specific primer pairs for verotoxin genes (*vtx1* and *vtx2*), intimin gene (*eaeA*), heat-stable enterotoxin A genes (*estA*-human and *estA*-porcine), heat-labile enterotoxin A (*eltA*), and invasion plasmid antigen H gene (*ipaH*) were used in detection of DEC pathotypes as previously described by Persson et al. [23] as shown in Table 1. The oligonucleotide primer sequences for the virulence factors and PCR product sizes. The PCR was performed using a Thermo-cycler (Eppendorf-Nethel-Hinz GmbH, Hamburg, Germany) with a reaction volume of 20 µL containing 1X of Solis BioDyne 5× FIREPol^®^ Master Mix with 12.5 mM MgCl_2_, primers mix, and 4 µL DNA template. The amplification condition comprised an initial denaturation at 95 °C for 15 min followed by 35 cycles of denaturation at 95 °C for 50 s, annealing at 57 °C for 40 s, elongation at 72 °C for 50 s, and then final elongation at 72 °C for 3 min. The PCR amplicons were visualized on 1.8% *w*/*v* agarose gel electrophoresis stained with ethidium bromide. The *E. coli* strain ATCC25922 was used as a negative control.

### 2.7. Antibiotic Susceptibility Test

The DEC isolates were tested for susceptibility to Ciprofloxacin (CPR), Tetracycline (TE), Ceftazidime (CAZ), Cefuroxime (CRX), Gentamicin (GEN), Cefixime (CXM), Ofloxacin (OFL), Augmentin (AUG), Nitrofurantoin (NIT) using Kirby–Bauer disk diffusion technique [24] on Mueller Hinton agar (Oxoid, UK) according to the Clinical and Laboratory Standards Institute (CLSI) guidelines. Following standard procedure, inhibition zone diameter breakpoints for each antibiotic were measured, and the susceptibility results were interpreted using CLSI guidelines [25]. *E. coli* (ATCC25922) was included as quality control organisms in antimicrobial susceptibility determination.

### 2.8. Over-Expression of Efflux Pump Activity in Multidrug Resistant (MDR) DEC Isolates

The DEC isolates resistant to three or more classes of antibiotics were classified as multidrug-resistant (MDR) [26]. MDR-DEC isolates were screened for phenotypic over-expression of efflux pump activity using the ethidium bromide (EtBr)-agar cartwheel method, as described by Martins et al. [27]. Briefly, over-night cultures of MDR DEC isolates were streaked on Trypticase Soy Agar (TSA) (Oxoid, UK) having 0.0 mg/L, 0.5 mg/L, 1.0 mg/L, 1.5 mg/L, and 2.0 mg/L of EtBr and incubated at 37 °C. The degree of fluorescence for each plate was examined under a UV transilluminator (Cleaver Scientific Ltd., Warwickshire, UK). Isolates without fluorescence at minimum concentration of EtBr were recorded to have over-expressed efflux pump activity, while those with fluorescence had no efflux pump activity.

### 2.9. Statistical Analysis

Descriptive statistic was used to describe the results of this research. Univariate analyses were performed to explore the association between social demographic factors, clinical manifestations, exposure sources, and the presence of DEC among children with diarrhea. The analyses were performed using the Chi-square test implemented in IBM SPSS version 20 (SPSS Inc. Chicago, IL, USA), and the level of significance was set at *p* < 0.05.

## 3. Results

### 3.1. Isolation of Presumptive DEC

A total of 228 diarrheal stool samples from children under 5 years attending or admitted in both private and public health facilities in Ogun State, Nigeria, were screened for DEC on selective agar, in which 1240 presumptive DEC isolates were obtained. In addition, 924 presumptive DEC isolates were obtained from 362 food sources (leftover household food = 18, fresh produce from markets = 252, and household drinking water = 92) and 183 wastewater samples from households and markets in proximity to the patients’ households.

### 3.2. Prevalence of Hetero-Pathogenic and DEC Pathotypes among the Diarrheic Children

A total of 139 isolates were confirmed to be DEC by multiplex PCR (Table 2, Figure S1a–e, Figure S2). The most prevalent pathotype was ETEC, with the isolates having the following virulent gene distribution: *estA-p* (69.1%), *estA-h* (9.4%), and *estA-p/estA-h* (8.6%), as shown in Table 2 The *eae* gene, which signifies EPEC, was found in 5% of the DEC isolates. Various combinations of virulent genes from two or more pathotypes, known as hetero-pathogenic DEC, were found in some of the DEC isolates. The most prevalent of these combinations were *estA-p/eae* (4.3%), indicative of the ETEC-EPEC heteropathotype, while four virulent genes combination of *vtx1/estA-h/estA-p*, *vtx2/estA-h*, *vtx1/vtx2/estA-h*, and *vtx1/vtx2/estA-p* representing the ETEC-VTEC hetero-pathotype. The EIEC-ETEC hetero-pathotype with the virulent gene combination of *ipaH/estA-h/estA-p* was also detected. However, the *eltA* gene was not detected in any of the isolates. The most prevalent DEC pathotype among the index cases was ETEC (21.05%), while the ETEC-EPEC (2.13%) was the most prevalent DEC hetero-pathotype. Overall, the prevalence of hetero-pathogenic and DEC pathotypes was 25.9, as presented in Table 3 (Figure S3).

### 3.3. Distribution of Virulence Genes and DEC Pathotypes in Various Food and Environmental Sources

In the food samples, the most prevalent pathotype was ETEC, with the isolates having the following virulent genes: *estA-h* (11.1%), *estA-p* (6.4%), and *estA-p/estA-h* (4.7%) (Table 4). The least prevalent single pathotypes were EPEC and EIEC, with both having a prevalence of 0.6% (Table 4). Furthermore, ETEC-VTEC (2.2%) was the most prevalent heteropathotype, while ETEC-EPEC-VTEC (0.3%) was the least prevalent (Table 4). Investigation into the specific food sources showed that fresh produce had the highest prevalence and distribution of hetero-pathogenic and DEC pathotypes, followed by drinking water and leftover food, as presented in Table 5. Conversely, in the wastewater samples, ETEC (2.2%) was the only DEC pathotype detected (Table 5). The distribution of DEC in the fresh farm produce is presented in Table 6, with the highest prevalence and the largest diversity of DEC pathotypes observed in cabbage.

### 3.4. Socio-Demographic and Risk Factors of DEC Pathotypes

The results of univariate risk factor analysis for the occurrence of DEC infection among diarrheic children under the age of five are presented in Table 7. DEC-infected children were more likely to experience loss of appetite (OR = 2.3, *p* = 0.012) and abdominal pain (OR = 2.4, *p* = 0.013) compared to diarrheic children with other etiologies. No statistically significant association (OR = 1.5, *p* = 0.37) was observed between DEC infection in males compared to females. Children with DEC infection were more likely to be exposed to street-vended foods (OR = 2.1, *p* = 0.04) than children with diarrhea from other causes.

### 3.5. Antimicrobial Susceptibility and Multidrug Resistance Profiles of Selected DEC Isolates

**Figure 1:**
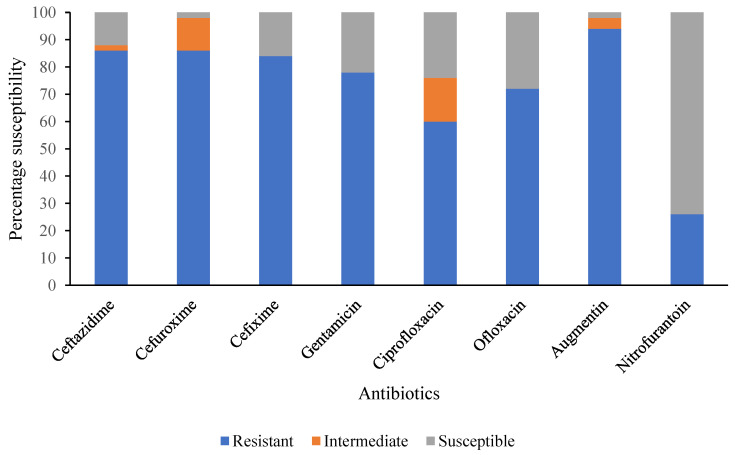
Summary of antimicrobial susceptibility pattern of selected DEC isolates from diarrheic children under 5 years in Ogun State, Nigeria.

The antimicrobial susceptibility profiles of the selected DEC isolates are summarized in Figure 1. Notably, very high resistance to commonly used antibiotics such as Augmentin (94%), Ceftazidime (86%), Cefuroxime (86%), and Cefixime (84%) was observed, while a relatively lower percentage was recorded for Nitrofurantoin (26%) (Figure 1). The majority of DEC pathotypes tested were MDR, as shown in Table 8. A total of 50 DEC isolates were analysed for antimicrobial resistance; 40 (80%) of the DEC can be categorised as multidrug-resistant (MDR) strains being resistant to more than three unrelated antimicrobial agents. However, the most predominant resistance phenotype (n = 19) was CAZ, CRX, GEN, CXM, OFL, AUG, and CPR. Furthermore, overexpression of efflux pump activity known to potentiate MDR phenotype was found in ETEC and EPEC isolates, as presented in Table 8.

## 4. Discussion

Diarrhea of bacterial etiology is often self-limiting, requiring no prior identification for patient treatment. However, in cases of prolonged or severe diarrhea (indicative of invasive infection), the identification of etiological agents becomes necessary for effective treatment [28]. The availability of surveillance and other epidemiological data is vital in the empirical management of and policy-making on preventing diarrheal diseases, including those of DEC etiology [29,30]. Regrettably, there is a paucity of such data in resource-limited countries like Nigeria due to the lack of sufficient functional laboratories and the limitations of currently used diagnostic techniques in routine laboratories. In Nigeria, data on the molecular epidemiology of DEC pathotypes in children under five years and their exposure environment is limited.

The current study revealed the relative prevalence and risk factors of hetero-pathogenic and diarrheagenic *E. coli* isolated from children under 5 years presented with diarrhea in hospitals situated in Ogun state. Outcomes from this study showed that DEC pathotypes were detectable in approximately 25.9% of the total diarrhea cases. This prevalence is within the prevalence range of DEC pathotypes earlier reported in Nigeria (18.4–73.8%) [7,17,31,32,33,34] and in Africa (7.4–86.5%) [35,36,37,38,39]. The observed variations among studies in DEC prevalence may be associated with differences in demography, geography, study population, and reservoir of DEC pathotypes. Still, the occurrence of DEC pathotypes is of clinical importance and underscores its significance as a major cause of infectious diarrhea in children in Ogun State, Nigeria.

Most significant and of great concern in the current study is the high prevalence of ETEC pathotype (21.05%), which was higher than previously reported cases (2.4–18.0%) in other regions in Nigeria [7,17,31,32,33,34]. Enterotoxigenic *E. coli* is known to harbor the heat-stable enterotoxin A (*estA*), which is further classified into two subtypes, known as *estA-p* and *estA-h*, because they were initially isolated from strains of pigs and human origin, respectively [40]. Heat-stable toxins producing ETEC isolated in this study are of public health significance and are commonly associated with severe diarrhea among children in low-income countries [41,42,43]. Interestingly, the high prevalence of *estA-p* and *estA-h* in the studied population and in fresh produce suggests environmental contamination with the possibility of a vibrant non-human reservoir of infection, which constitutes an exposure source for children in these communities.

The presence of the *eae* (*E. coli* attachment effacement) gene within the studied population is indicative of EPEC [44]. The *eae* gene encodes an outer membrane protein, intimin, which mediates adherence of these pathotypes to enterocytes, thereby starting the effacement of gastric epithelial–cell microvilli [44,45,46]. EPEC has been implicated as a leading cause of infantile diarrhea in developing countries [6,47]. The occurrence of EPEC pathotype in Nigeria is not unusual; however, the prevalence in this study (0.44%) was lower than that previously reported (1.8–15.0%) in other parts of the country [7,17,31,32,33,34]. Age category, study location, and study period may contribute to this observed discrepancy. Humans and animals are reservoirs for EPEC [6]. So, the absence of potential risk factors (such as the practice of open defecation) that could mediate transmission from the human reservoir may also be a possible reason for a lower prevalence.

Hetero- or hybrid pathotypes are terms used to describe new combinations of virulence factors among DEC [13]. In 2015, an EPEC expressing the ETEC heat-labile toxin was observed in India [15]. The same combinations in the present study were reported in a study done in South Africa [48]. Approximately 55% (6/11) of the ETEC/EPEC hybrid strains in the present study were detected in diarrheic children. In addition, the relative prevalence of EPEC/ETEC hetero-pathogenic pathotype was similar to that reported previously in the northern part of Nigeria [7,15,33]. The *vtx1* and *vtx2* genes occurred as VTEC/ETEC hetero-pathotypes with a prevalence of 1.3%, which was lower than a single VTEC pathotype earlier reported in other studies [17,31,34,49]. The presence of *estA/vtx1/vtx2* producing hetero-pathogenic VTEC/ETEC pathotype could increase the progression of clinical manifestations such as hemolytic uremic syndrome (HUS), thrombotic thrombocytopenic purpura, and hemorrhagic colitis [50,51,52] that are commonly associated with single pathotype infection. Likewise, the detection of the ipaH/estA gene indicative of the EIEC/ETEC hetero-pathotype with a prevalence of 0.44% was reported in this study. Previous studies have implicated a single EIEC pathotype as the etiology of different outbreaks of bacillary dysentery across the globe [45,53,54]; however, there is a paucity of scientific data on the prevalence or detection of EIEC/ETEC hetero-pathotype from human cases. The low prevalence of hetero-pathotype suggests a possible low but emerging epidemiological pattern in Nigeria. The genomic plasticity of *E. coli* allows for the emergence of new hybrid strains, which could result in severe outbreaks, heightened levels of pathogenesis, and immune evasion [7,13,55,56,57,58,59,60,61]. Heteropathotype infection could also increase the transmission of resistant genes, which may complicate the available treatment options for DEC infections.

In the food samples, the wide distribution of various hetero-pathogenic and DEC pathotypes in the fresh produce samples suggests these food types are an important source of DEC infection in the studied population. This is anticipated because fresh produce is often served raw to children as a form of nutritional supplement and undergoes minimal processing. Moreover, fresh produce is often grown with organic manure laden with a high number of microorganisms [62,63,64,65,66]. The presence of DEC pathotypes in drinking water and leftover food samples further implicates these sources as being probable sources of infection. Conversely, only ETEC was found in the wastewater samples, indicating this as a likely reservoir.

Generally, episodes of diarrhea are often accompanied by clinical manifestations such as watery diarrhea, vomiting, fever, loss of appetite as well as abdominal pain [67]. In this study, diarrheic children with associated DEC pathotypes showed these clinical manifestations. These symptoms were not related to specific DEC pathotypes, which is consistent with other studies describing that infections with any DEC pathotypes are indistinguishable based on clinical manifestations [30,68]. The age distribution analysis of the DEC pathotype in the current research agrees with previous findings that the incidence of DEC reduces with an increase in age [7,33]. The UNICEF/WHO encourages exclusive breastfeeding for the first 6 months of a child’s life [69] as a possible means of reducing the incidence of diarrhea. Despite this advocacy, DEC was still detectable among children who were exclusively breastfed. In addition, the incidence of DEC among the other feeding type sub-groups is indicative of a hygiene-related problem. Analysis of the associated risk factors of DEC infection revealed that diarrheic children exposed to street-vended foods were more likely to be infected with DEC infection than children fed homemade foods. This agrees with the findings of Estrada-Garcia et al. [70], Islam et al. [71], and Ugboko et al. [72] that DEC is more often associated with street-vended foods in developing countries. A possible explanation for this might be the prolonged exposure of these foods to open air and the likelihood of inadequate quality assurance measures in the production process. Such findings call for extra caution on the part of parents and caregivers to selectively buy ready-to-eat foods from vendors with good hygiene. The occupational status of caregivers does not correlate with the frequency of DEC infections. Hence, consumption of street-vended foods and contaminated fresh produce could be the drivers of DEC infection in children in Ogun State, Nigeria. These results, notwithstanding, underscore the significance of non-human reservoirs as sources of infection, as other studies had previously reported the presence of DEC in water and other non-human reservoirs within the state [73,74,75,76]. Provision of a potable water supply is still recommended, and in cases when such is not available, the boiling of drinking water is not negotiable in the prevention of diarrhea due to DEC.

Accessibility, availability, and timeliness of interventions such as supportive therapy, anti-secretory drug therapy, and specific antimicrobial chemotherapy are critical to reducing diarrhea-associated morbidity and mortality, especially in children under 5 years found in rural and resource-limited populations [74]. Administration of ORS and zinc is considered the first line of treatment in the management of diarrhea infection in children under 5 years [3]. In the current study, ORS was only administered in 50.9% of all the DEC-associated diarrhea cases. This implies that a good number of the caregivers knew what actions to perform during episodes of diarrhea. However, there is a need to create more awareness of the use of ORS in the management of diarrhea, especially in rural communities.

DEC infections are often self-limiting, requiring no interventions [6,7]. Conversely, infections with ETEC, EPEC, and EIEC pathotypes can result in persistent, severe, and invasive diseases that may require the use of antibiotics for effective treatment [6]. Antibiotics such as Cephalosporin, Fluoroquinolones, Penicillin (Augmentin), and sulfonamides are among the commonly used antibiotics for the management of bacterial diarrhea in Nigeria. In recent years, the alarming rising incidence of MDR DEC, especially extended-spectrum β-lactamase (ESBL) and carbapenemase-producing strains is of global public health significance [6]. The inappropriate usage and lack of antibiotic stewardship in the management of diarrheal diseases in children is a problem in Nigeria [77]. In the present study, representatives of the isolated DEC pathotypes subjected to antimicrobial susceptibility assay showed different levels of susceptibility and resistance to all the tested antibiotics, with Cephalosporin and Augmentin having the highest observed resistance. Resistance to the tested antibiotics, as well as the observed multidrug resistance to the Cephalosporin–Aminoglycoside–Penicillin class of antibiotics in this study, is suggestive of extended-spectrum β-lactamase (ESBL) activity, which is an emerging problem in the treatment of DEC infection in children under 5 years in Nigeria [78]. Resistance to some of these commonly used antibiotics was also found in Ethiopia [6], South Africa [79], and Gabon [80]. Investigation into the probable cause of observed multidrug resistance revealed over-expression of efflux pump activity among some of the ETEC and EPEC pathotypes. Efflux pump activity has been shown to potentiate the evolution of antibiotic resistance among bacteria isolates [81].

## 5. Conclusions

Enterotoxigenic *E. coli* (ETEC) was found to be the predominant pathotype implicated in cases of childhood diarrhea in the studied population. The detection of DEC in fresh farm produce is a clear indication of food safety risk, especially among children. Consumers of farm products should wash them thoroughly with potable water before consumption to reduce the risk of infection with DEC. Continuous surveillance of DEC pathotypes as part of the routine surveillance program is highly recommended. Further studies to increase our understanding of the implication of DEC hetero-pathotypes on disease progression and severity are recommended. Information from studies of this nature will further enrich empirical treatment, especially in locations where such diagnosis cannot be routinely performed. Antibiotic stewardship should be given priority in LMIC to stem down cases of multidrug resistance. Additionally, the persistence of the DEC pathotype in this study calls for the requirement of re-appraisal of current diarrhea prevention programs available in the country. Continuous education on diarrhea prevention and care for every stakeholder involved in pediatric care is, thereby, recommended.

## Figures and Tables

**Table 1:** Oligonucleotide primers used for PCR amplification of the targeted virulence genes in isolated DEC [23].

Primer	Gene Target	Virulence Factor/Gene	Primer Sequence (5′-3′)	Final Conc. (µM)	Amplicon Size (bp)
StFh	Human *estA*	ST1h	TTTCGCTCAGGATGCTAAACCAG	0.4	151
StRh			CAGGATTACAACACAATTCACAGCAGTA		
StFp	Porcine *estA*	ST1p	CTTTCCCCTCTTTTAGTCAGTCAACTG	0.4	160
StRp			CAGGATTACAACAAAGTTCACAGCAG		
PS3	*vtx1*	VT1	GTTTGCAGTTGATGTCAGAGGGA	0.25	260
PS4			CAACGAATGGCGATTTATCTGC		
PS5	*Eae*	Intimin	GGYCAGCGTTTTTTCCTTCCTG	0.15	377
PS6			TCGTCACCARAGGAATCGGAG		
PS7	*vtx2*	VT2	GCCTGTCGCCAGTTATCTGACA	0.5	420
PS8			GGAATGCAAATCAGTCGTCACTC		
PS9	*eltA*	LTI	AAACCGGCTTTGTCAGATATGATGA	0.45	479
PS10			TGTGCTCAGATTCTGGGTCTCCT		
PS11	*ipaH*	IpaH	TTGACCGCCTTTCCGATACC	0.1	647
PS12			ATCCGCATCACCGCTCAGAC		

**Table 2:** Distribution of virulence genes among DEC isolated (*n* = 139) in diarrheic children under 5 years in Ogun State, Nigeria.

Virulence Genes							DEC Pathotypes		Hetero-Pathogenic DEC Pathotypes		
*ipaH*	*vtx1*	*vtx2*	*eae*	*eltA*	*estA-h*	*estA-p*	ETEC	EPEC	ETEC/EPEC	ETEC/EIEC	ETEC/VTEC
-	+	-	-	-	+	+	-	-	-	-	1 (0.7) *
-	-	+	-	-	+	-	-	-	-	-	1 (0.7)
-	+	+	-	-	+	-	-	-	-	-	1(0.7)
-	+	+	-	-	-	+	-	-	-	-	1 (0.7)
-	-	-	+	-	-	+	-	-	6 (4.3)	-	-
+	-	-	-	-	+	+	-	-	-	1 (0.7)	-
-	-	-	+	-	-	-	-	7 (5.0)	-	-	-
-	-	-	-	-	+	-	13 (9.4)	-	-	-	-
-	-	-	-	-	-	+	96 (69.1)	-	-	-	-
-	-	-	-	-	+	+	12 (8.6)	-	-	-	-
TOTAL							121 (87.1)	7 (5.0)	6 (4.6)	1 (0.7)	4 (2.9)

- Represents absence of virulence genes; + Represents presence of virulence genes; * % Occurrence of DEC pathotype; ETEC—enterotoxigenic *E. coli*; VTEC—Verotoxigenic *E. coli*; EPEC—enteropathogenic *E. coli*; EIEC—enteroinvasive *E. coil*.

**Table 3:** Prevalence of DEC pathotypes among cases (n = 228) of diarrheic children under 5 years in Ogun State, Nigeria.

Pathotypes	Number of Child Cases	% Prevalence
ETEC	49	21.05
EPEC	1	0.44
DEC Hetero-pathotypes		
VTEC-ETEC	3	1.32
ETEC-EPEC	5	2.13
ETEC-EIEC	1	0.44
Total	59	25.88

ETEC—enterotoxigenic *E. coli*; VTEC—Verotoxigenic *E. coli*; EPEC—enteropathogenic *E. coli*; EIEC—enteroinvasive *E. coli.*

**Table 4:** Distribution of virulence genes among DEC pathotypes isolated from food and water sources in Ogun State, Nigeria.

Virulence Genes							Food Sources (*n* = 362)							
							DEC Pathotypes				Hetero-Pathogenic DEC			
*ipaH*	*vtx1*	*vtx2*	*eae*	*eltA*	*estA-h*	*estA-p*	ETEC	VTEC	EPEC	EIEC	ETEC-VTEC	ETEC-EIEC	ETEC-EPEC	ETEC-EPEC-VTEC
-	-	-	-	-	+	-	40 (11.1) *	-	-	-	-	-	-	-
-	-	-	-	-	-	+	23 (6.4)	-	-	-	-	-	-	-
-	-	-	-	-	+	+	17 (4.7)	-	-	-	-	-	-	-
-	+	+	-	-	-	-	-	2 (0.6)	-	-	-	-	-	-
-	+	-	-	-	-	-	-	4 (1.1)	-	-	-	-	-	-
-	-	+	-	-	-	-	-	1 (0.3)	-	-	-	-	-	-
+	-	-	-	-	-	-	-	-	-	2 (0.6)	-	-	-	-
-	-	-	+	-	-	-	-	-	2 (0.6)	-	-	-	-	-
-	+	-	-	-	+	+	-	-	-	-	2 (0.6)	-	-	-
+	-	-	-	-	+	+	-	-	-	-	-	2 (0.6)	-	-
-	+	-	-	-	-	+	-	-	-	-	1 (0.3)	-	-	-
-	-	-	+	-	+	-	-	-	-	-	-	-	2 (0.6)	-
-	+	-	-	-	+	-	-	-	-	-	5 (1.4)	-	-	-
-	-	-	+	-	+	+	-	-	-	-	-	-	1 (0.3)	-
-	+	-	+	-	-	+	-	-	-	-	-	-	-	1 (0.3)
TOTAL							80 (22.1)	7 (1.9)	2 (0.6)	2 (0.6)	8 (2.2)	2 (0.6)	3 (0.8)	1 (0.3)

- Represents absence of virulence genes; + Represents presence of virulence genes; * % Occurrence of DEC pathotype; ETEC—enterotoxigenic *E. coli*; VTEC—Verotoxigenic *E. coli*; EPEC—enteropathogenic *E. coli*; EIEC—enteroinvasive *E. coli*.

**Table 5:** Distribution of hetero-pathogenic and DEC pathotypes among various food and environmental sources in Ogun State, Nigeria.

Sources		Positive Samples for DEC Pathotypes							
		ETEC	EPEC	VTEC	EIEC	ETEC-VTEC	ETEC-EIEC	ETEC-EPEC	ETEC-EPEC-VTEC
Food	Leftover food (*n* = 18)	1 (5.6) *	-	-	-	1 (5.6)	-	-	-
	Fresh produce (*n* = 252)	73 (29.0)	2 (0.8)	5 (2.0)	2 (0.8)	6 (2.4)	2 (0.8)	3 (1.2)	1 (0.4)
	Drinking water (*n* = 92)	2 (2.2)	-	2 (2.2)	-	1 (1.1)	-	-	-
Wastewater (*n* = 183)		4 (2.2)	-	-	-	-	-	-	-
Total		80	2	7	2	8	2	3	1

* % Occurrence of DEC pathotype; ETEC—enterotoxigenic *E. coli*; VTEC—Verotoxigenic *E. coli*; EPEC—enteropathogenic *E. coli*; EIEC—enteroinvasive *E. coli*.

**Table 6:** Distribution of hetero-pathogenic and DEC isolates in fresh farm produce in Ogun State, Nigeria.

			Number of Positive Samples (% Positive) *								
Sources		Number of Samples	ETEC	EPEC	VTEC	EIEC	ETEC-VTEC	ETEC-EIEC	ETEC-EPEC	ETEC-EPEC-VTEC	Total DEC
Fresh farm produce	Cabbage	36	17 (47.2) *	1 (2.8)	2 (5.6)	1 (2.8)	2 (5.6)	1 (2.8)	1 (2.8)	1 (2.8)	26 (72.2)
	Carrot	36	15 (41.7)	-	1 (2.8)	1 (2.8)	2 (5.6)	1 (2.8)	-	-	20 (55.6)
	Cucumber	36	11 (30.6)	-	1 (2.8)	-	1 (2.8)	-	-	-	13 (36.1)
	Watermelon	36	6 (16.7)	-	-	-	-	-	-	-	6 (16.7)
	Pineapple	36	10 (27.8)	-	-	-	-	-	1 (2.8)	-	11 (30.6)
	Lettuce	36	9 (25)	-	1 (2.8)	-	1 (2.8)	-	-	-	11 (30.6)
	Pawpaw	36	5 (13.9)	1 (2.8)	-	-	-	-	1 (2.8)	-	7 (19.4)
Total		252	73 (29.0)	2 (0.8)	5 (2.0)	2 (0.8)	6 (2.4)	2 (0.8)	3 (1.2)	1 (0.4)	

* percentage positive of DEC pathotypes.

**Table 7:** Socio-demographic and risk factors predictor of DEC pathotypes among cases of diarrheic children under 5 years in Ogun State, Nigeria.

Variable	Total No.	Cases with DEC	Percentage (%)	OR (95% CI)	*p* Value
Gender					
Female	110	25	22.73	1	
Male	118	34	28.81	1.5 (0.8–2.5)	0.37
Age (year)					
2–5	131	31	23.66	1	
Less than 2	97	28	28.87	0.8 (0.4–1.4)	0.46
Fever					
Absent	98	21	21.43	1	
Present	130	38	29.23	1.5 (0.8–2.8)	0.24
Vomiting					
No	143	37	25.87	1	
Yes	85	22	25.88	1.0 (0.5–1.9)	1
Appetite					
Yes	134	33	24.63	1	
No	94	26	27.66	2.3 (1.2–4.1)	0.012 *
Nausea					
No	193	49	25.39	1	
Yes	35	10	28.57	1.2 (0.5–2.6)	0.85
Abdominal pain					
Absent	169	36	21.3	1	
Present	59	23	38.98	2.4 (1.3–4.5)	0.013 *
ORS before Hospital presentation			23.02		
No	126	29	29.41	1	
Yes	102	30		1.4 (0.8–2.5)	0.35
Self-prescribed antibiotic			25.73		
No	206	53	27.27	1	
Yes	22	6		1.1 (0.4–2.9)	1
Site of enrollment					
School/daycare	167	18	10.78	1	
Others	60	41	68.33	1.3 (0.7–2.5)	0.51
Other siblings with similar symptoms			26.92		
Yes	26	7	25.74	1	
No	202	52		1.1 (0.42–2.7)	1
Feeding Type					
Breastfeeding	38	11	28.95	1	
Breastfeeding + Infant formula	38	9	23.68	0.8 (0.3–2.1)	
Breastfeeding + Infant formula + Solid food	53	16	30.19	1.1 (0.4–2.7)	
Infant formula + weaning food	27	7	25.93	0.9 (0.3–2.6)	
Solid food	71	16	22.54	0.7 (0.3–1.8)	
Street food consumption					
Absent	168	37	22.02	1	
Present	60	22	36.67	2.1 (1.1–3.9)	0.04 *
Type of toilet used					
Water closet	194	51	26.29	1	
Pit Latrine	11	4	36.36	1.6 (0.5–5.7)	
Potty	23	4	17.39	0.6 (0.2–1.8)	
Source of drinking water					
Sachet water	68	8	11.76	1	
Bottled water	41	12	29.27	0.6 (0.1–2.4)	
Private well/borehole	107	26	24.3	1.2 (0.3–5.4)	
Community tap	10	3	30	0.9 (0.2–3.6)	
Occupation of caregiver					
Merchant/trader	104	24	23.08	1	
Artisan/craftsman	42	7	16.67	0.5 (0.2–1.2)	
Office worker	54	15	27.78	1.1 (0.6–2.3)	
Teacher	10	2	20	0.6 (0.1–3.1)	
Housewife	16	1	6.25	0.6 (0.2–2.1)	

* % Occurrence of DEC pathotype.

**Table 8:** Antimicrobial and multidrug resistance (MDR) profile of selected DEC isolated from diarrheic children under 5 years in Ogun State, Nigeria.

		DEC Pathotypes Isolates			
		ETEC, *n* = 40	EPEC, *n* = 6	ETEC- VTEC, *n* = 3	ETEC- EIEC, *n* = 1
Single antimicrobial resistance phenotype	CAZ	34 (85) *	6 (100)	3 (100)	1 (100)
	CRX	34 (85)	5 (83)	3 (100)	1 (100)
	GEN	31 (77.5)	5 (83)	2 (66.7)	1 (100)
	CXM	34 (85)	5 (83)	2 (66.7)	1 (100)
	OFL	28 (70)	5 (83)	2 (66.7)	1 (100)
	AUG	38 (95)	5 (83)	3 (100)	1 (100)
	NIT	12 (30)	1 (16.7)	-	-
	CPR	23 (57.5)	5 (83)	2 (66.7)	1 (100)
MDR phenotype	CAZ, CRX, GEN, CXM, OFL, AUG, NIT, CPR,	10 (25)	1 (16.7)	-	-
	CAZ, CRX, GEN, CXM, OFL, AUG, CPR	13 (32.5)	3 (50)	2 (66.7)	1 (100)
	CAZ, CRX, GEN, CXM, OFL, AUG	-	1 (16.7)	-	-
	CAZ, CRX, CXM, OFL, AUG, CPR,	1 (2.5)	-	-	-
	CAZ, CRX, GEN, CXM, AUG	8 (20)	-	-	-
Efflux pump expression	Expressed	9 (22.5)	2 (33.3)	-	-
	Not expressed	23 (57.5)	3 (50)	3 (100)	1 (100)

* Percentage (%) occurrence of DEC pathotype.

## Data Availability

Data are available on request.

## Supplementary Materials

The supporting information can be downloaded at https://www.mdpi.com/article/10.3390/pathogens12111358/s1: Figure S1a–e: Agarose gel electrophoresis image of multiplex PCR amplicon of DEC isolates; Figure S2: Growth of presumptive DEC colonies on Sorbitol MacConkey Agar (A-Sorbitol fermenter and B—Non Sorbitol fermenter); Figure S3: Over expression of efflux pump assay for multidrug resistant DEC isolates.
