# Supplementary material for: Molecular Characterization of Hetero-Pathogenic and Diarrheagenic Escherichia coli Pathotypes in Diarrheic Children under Five Years and Exposure Environment in Ogun State, South-West Nigeria
Source: Pathogens. 2023 Nov 15;12(11):1358. doi: 10.3390/pathogens12111358 (PMC10675616; doi:10.3390/pathogens12111358)
Supplement: Supplementary file 1 [file pathogens-12-01358-s001.zip › pathogens-2677739-supplementary.pdf]

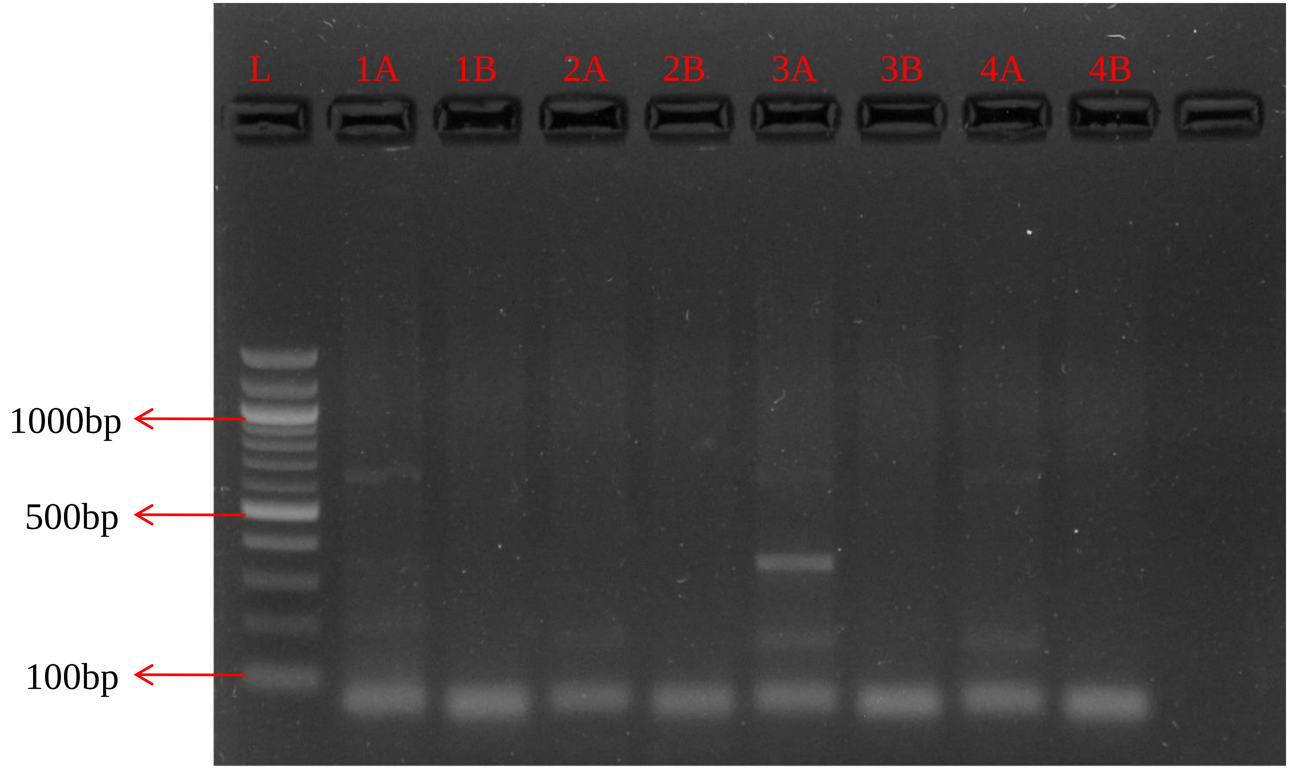

A. Human *estA* (StFh and StRh primers) – 151bp
vtx1 – 260bp
vtx2 – 420bp
ipaH – 647bp

B. Porcine *estA* (StFp and StRp primers) – 160bp
eae – 377bp
eltA – 479bp

1 – FH21E (ipaH)
2 – FH24E
3 – SH 70E (Human *estA*, *vtx1*, *ipaH*)
4 – FH25E (Human *estA*, *ipaH*)

Figure S1a: Agarose gel electrophoresis image of multiplex PCR amplicon of DEC isolates. L: 100bp molecular ladder. Sample codes: 1A-4B

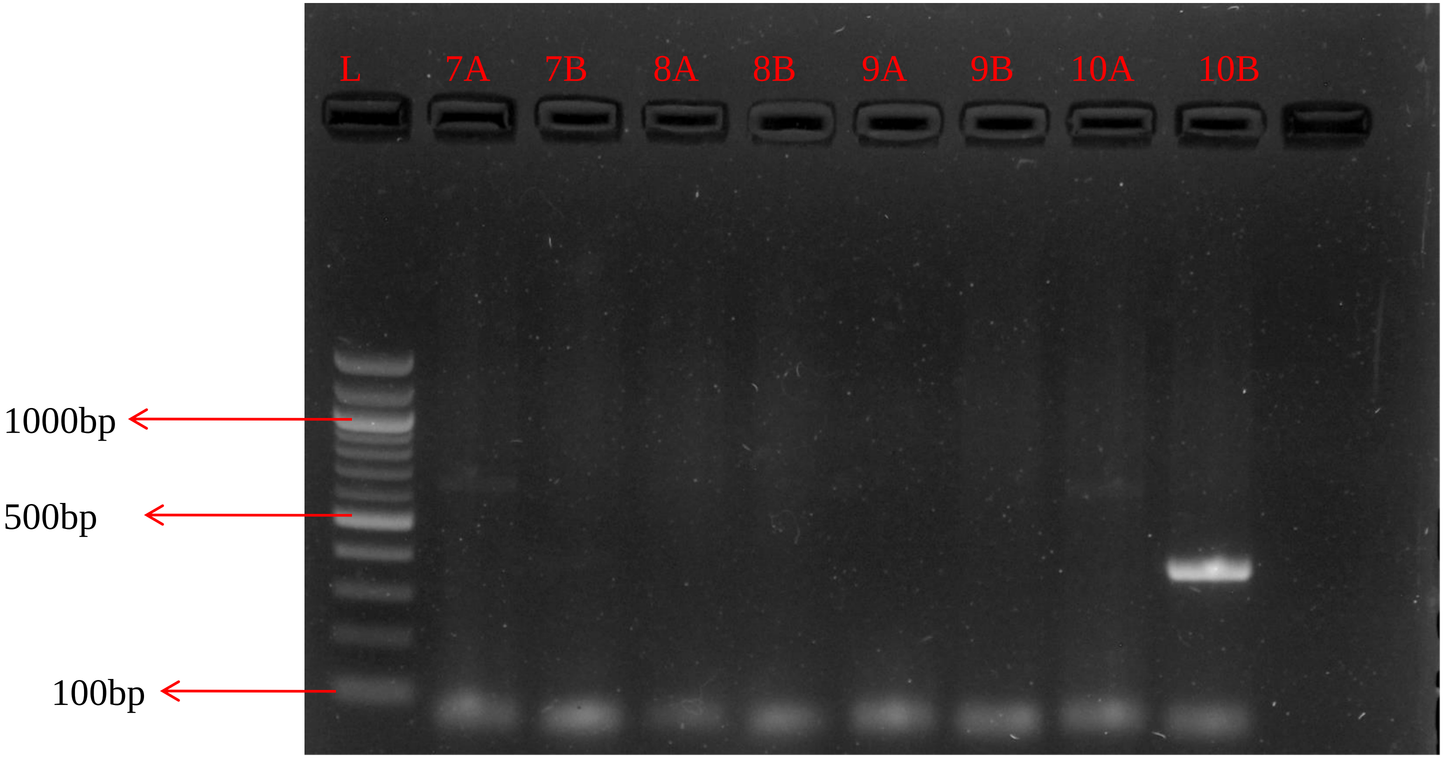

A. Human *estA* (StFh and StRh primers) – 151bp
vtx1 – 260bp
vtx2 – 420bp
ipaH – 647bp

B. Porcine *estA* (StFp and StRp primers) – 160bp
eae – 377bp
eltA – 479bp

7 – FH23E (ipaH)
8 – FH22E
9 – SH 73E
10 – SH74E (ipaH ,*eae)*

Figure S1b: Agarose gel electrophoresis image of multiplex PCR amplicon of DEC isolates. L: 100bp molecular ladder. Sample codes: 7A-10B

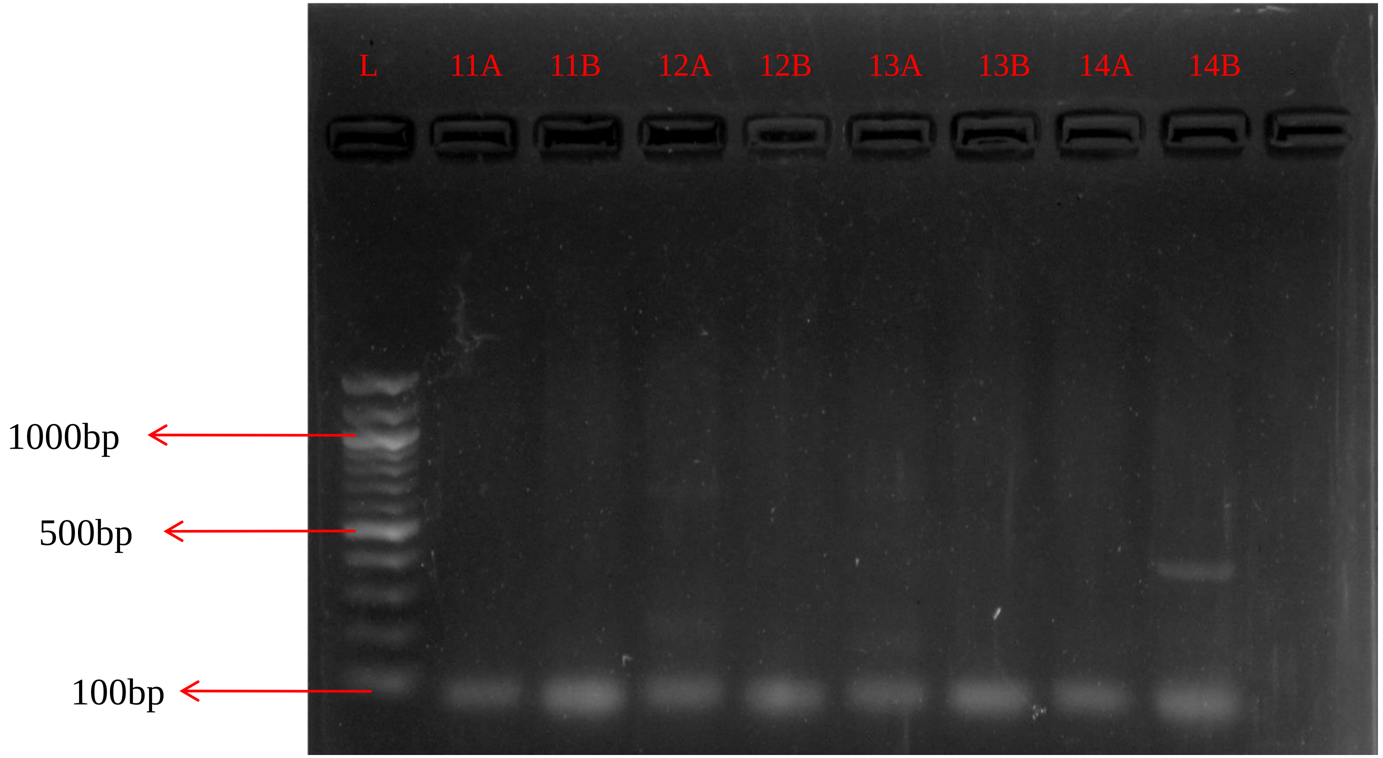

A. Human *estA* (StFh and StRh primers) – 151bp
vtx1 – 260bp
vtx2 – 420bp
ipaH – 647bp

B. Porcine *estA* (StFp and StRp primers) – 160bp
eae – 377bp
eltA – 479bp

11 – SH68E
12 – SH65E (Human *estA* , *ipaH*)
13 – SH61E
14 – SH67E *(eae)*

Figure S1c: Agarose gel electrophoresis image of multiplex PCR amplicon of DEC isolates. L: 100bp molecular ladder. Sample codes: 11A-14B

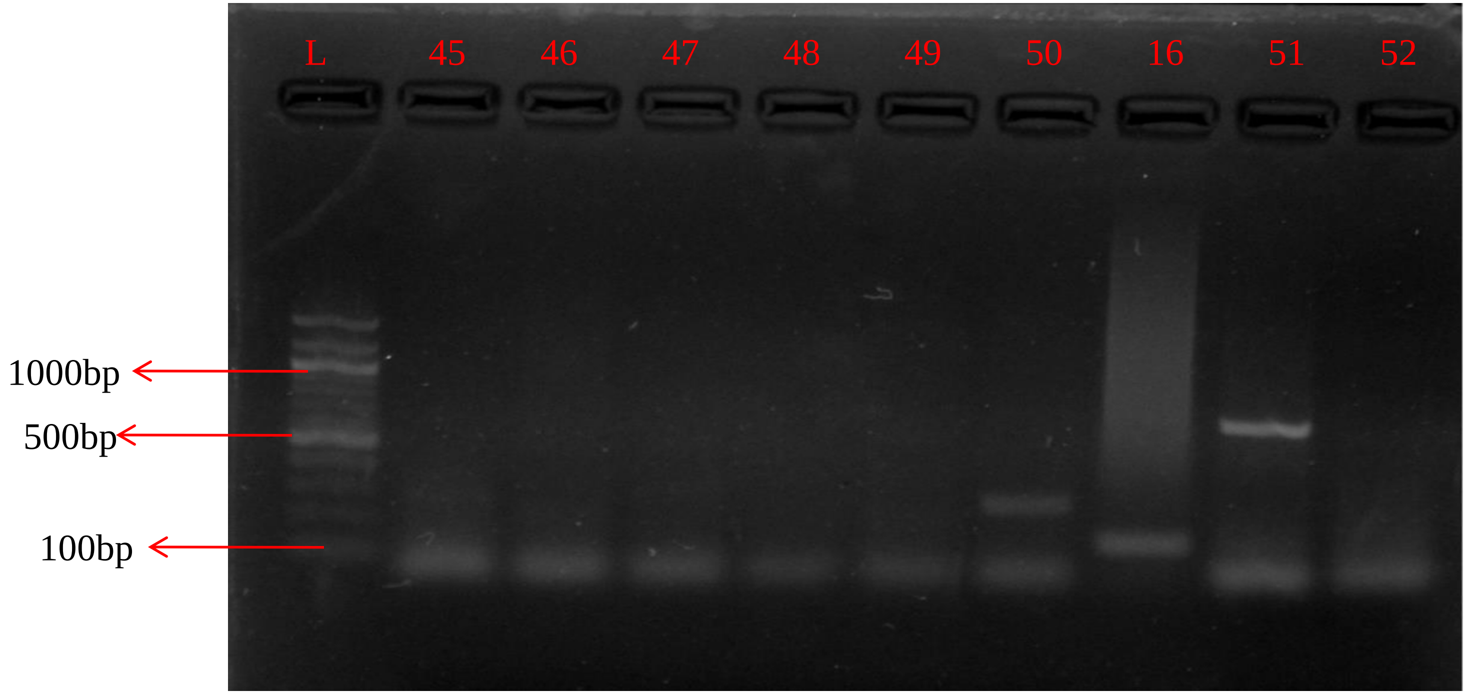

A. Human *estA* (StFh and StRh primers) – 151bp
vtx1 – 260bp
vtx2 – 420bp
ipaH – 647bp

B. Porcine *estA* (StFp and StRp primers) – 160bp
eae – 377bp
eltA – 479bp

45– SH90E
46 – GH4E
47 – SH93E
48 –SH92E
49 –SH4A
50 –OF1A (*vtx1)*
16 –SH100E (Human *estA*)
51 –FH11E (*ipaH*)
52 –FH12E

Figure S1d: Agarose gel electrophoresis image of multiplex PCR amplicon of DEC isolates. L: 100bp molecular ladder. Sample codes: 45-52

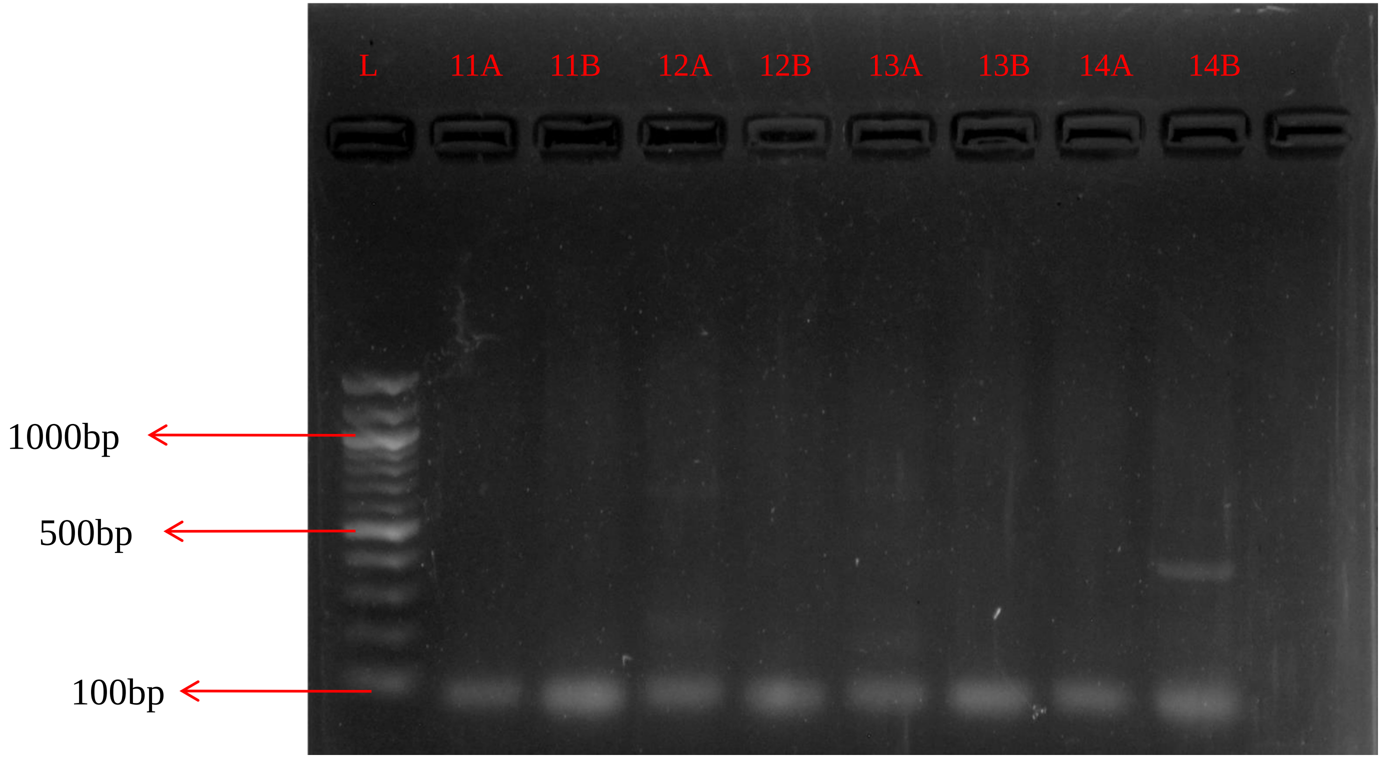

A. Human *estA* (StFh and StRh primers) – 151bp
vtx1 – 260bp
vtx2 – 420bp
ipaH – 647bp

B. Porcine *estA* (StFp and StRp primers) – 160bp
eae – 377bp
eltA – 479bp

11 – SH68E
12 – SH65E (Human *estA* , *ipaH*)
13 – SH61E
14 – SH67E *(eae)*

Figure S1e: Agarose gel electrophoresis image of multiplex PCR amplicon of DEC isolates. L: 100bp molecular ladder. Sample codes: 1A-4B

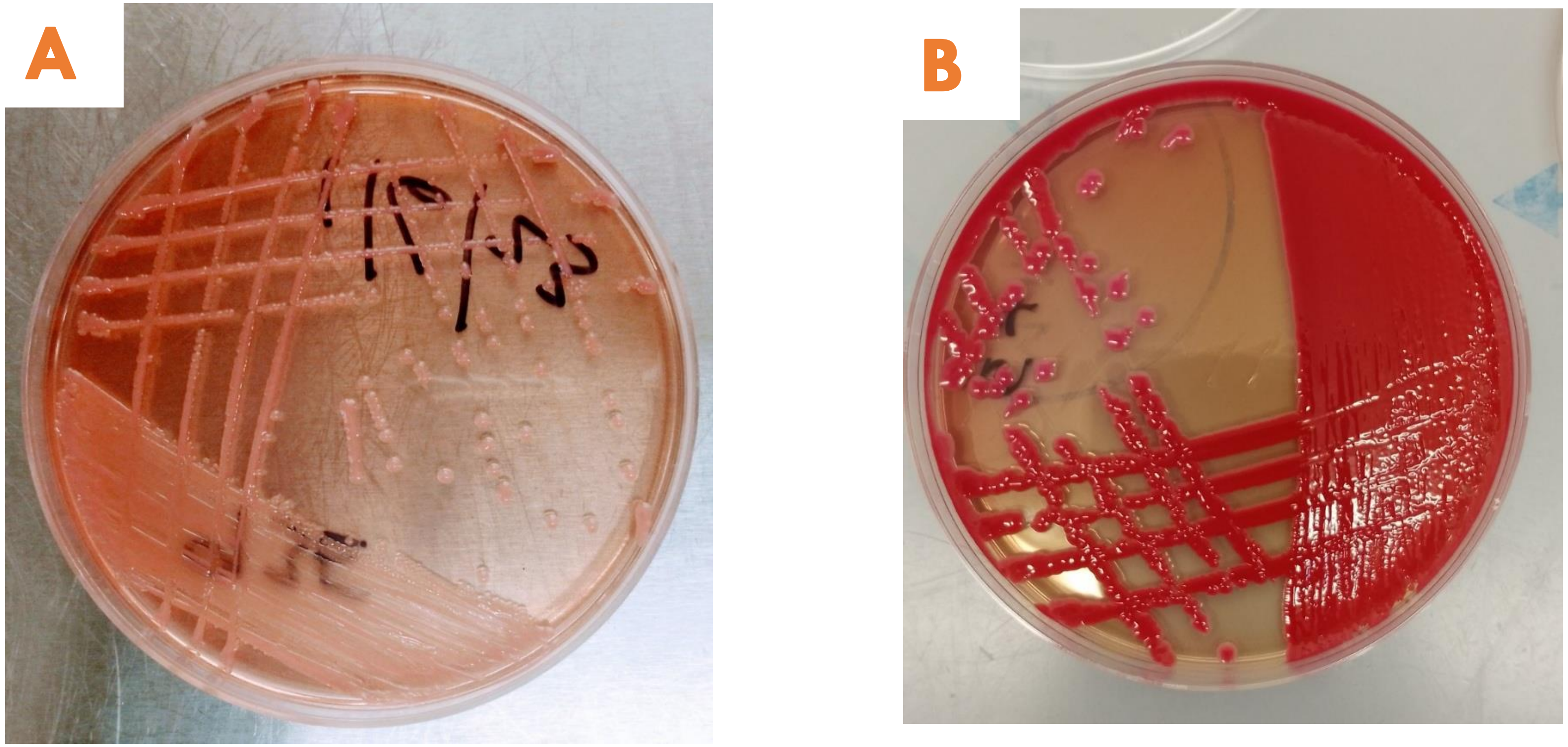

Figure S2: Growth of presumptive DEC colonies on Sorbitol MacConkey Agar (A-Sorbitol fermenter and B- Non Sorbitol fermenter)

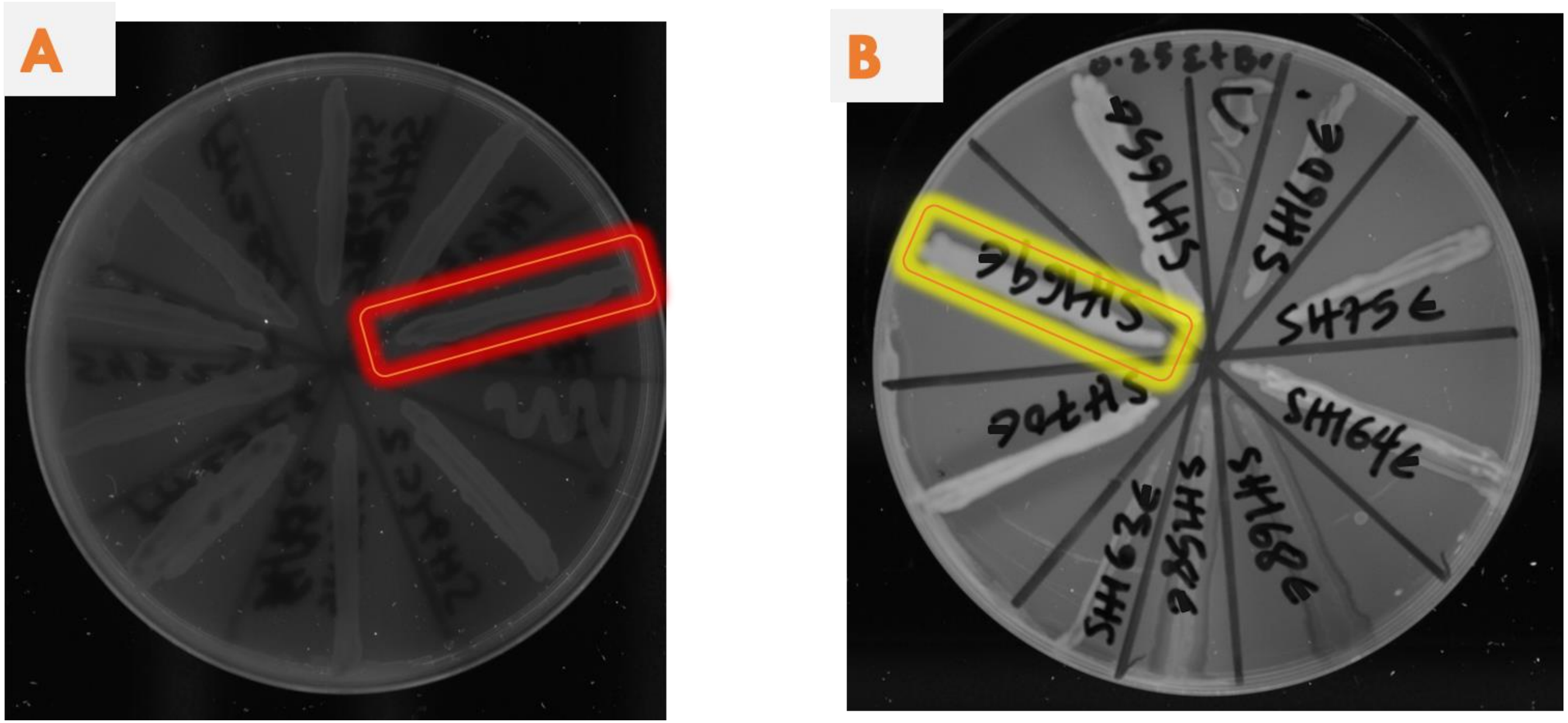

Figure S3: Over expression of efflux pump assay for multidrug resistant DEC isolates. A (Red box): over expression of efflux pump activity. B (Yellow box): No efflux pump activity
